# The Choice of Procedure in Loose Kidney

**Published:** 1907-04

**Authors:** 


### THE CHOICE OF PROCEDURE IN LOOSE KIDNEY.

Robert T. Morris, M. D., claims that both too much and too little operating has been done for loose kidney. Ten years ago operations for this condition were done indiscriminately. Loose kidney is found in ten per cent. of all women who come into the office, but not all of these need an operation. The following classification of loose kidney cases is given: (1) Loose kidney present, but causing no disturbance. (2) Patients with minor psychoses associated with, but not dependent upon the presence of loose kidney. (3) Patients with minor or major psychoses precipitated or intensified by the presence of loose kidney. (4) Patients with various reflex gastro-intestinal disturbance, depending upon the influence of loose kidney through the large sympathetic ganglia. These cases are the ones that respond best to surgery. (5) Patients with direct local mechanical results from influence of loose kidney. These cases give the cleanest cures after operation. (6) Patients in whom the loose kidney is only a part of a panptosis of the abdominal viscera. In these cases the other visceral ptoses must be corrected, as well as the kidney. The treatment consists of abdominal supporters for some of the cases, and operation for others. The operation consists of a combination of operations having for its object the hanging up of the kidney in a simple way, besides obliterating the retroperitoneal pouch below, by gauze packing which stimulates the formation of granulation tissue and fixes the nephro-colic ligament. —American Journal of Surgery, January, 1907.

